# Investigating the distribution of the Yangtze finless porpoise in the Yangtze River using environmental DNA

**DOI:** 10.1371/journal.pone.0221120

**Published:** 2019-08-09

**Authors:** Yongkai Tang, Yunsheng Wu, Kai Liu, Jianlin Li, Hongxia Li, Qin Wang, Juhua Yu, Pao Xu

**Affiliations:** 1 Key Laboratory of Freshwater Fisheries and Germplasm Resources Utilization, Ministry of Agriculture, Freshwater Fisheries Research Center, Chinese Academy of Fishery Sciences, Wuxi, China; 2 Wuxi Fisheries College, Nanjing Agricultural University, Wuxi, China; University of Hyogo, JAPAN

## Abstract

Determining the distribution of the Yangtze finless porpoise (*Neophocaena asiaeorientalis asiaeorientalis*, YFP) in the Yangtze River has to date relied on traditional visual and counting methods, but such field surveys are time-consuming and expensive. Analyses using environmental DNA (eDNA) to investigate the presence and range of endangered aquatic species have proven to be more economical and effective detection methods, and are a non-invasive approach to sampling. A challenge of relying on eDNA for YFP monitoring is that the Yangtze River is characterized by high turbidity and a strong current. Here, we used an eDNA-based approach to estimate the presence of YFP at 18 sites in the Yangtze River in August 2017 and at an additional 11 sites in January 2018. At each sampling site, we filtered six 1 L water samples with 5 µm pore size filter paper and quantified the amount of YFP eDNA in each water sample using quantitative real-time polymerase chain reaction (qPCR). In addition, YFP eDNA was successfully detected in locations where we visually observed YFP, as well as in locations where YFP were not observed directly. We found that our eDNA-based method had higher detection rates than traditional field survey methods. Although YFP was visually observed in the Yangtze River in winter, water samples collected during the summer contained significantly higher YFP eDNA than winter water samples. Our results demonstrate the potential effectiveness of eDNA detection methods in determining the distribution of YFP in the Yangtze River.

## Introduction

Yangtze finless porpoise (*Neophocaena asiaeorientalis asiaeorientalis*, YFP), a prominent species of aquatic conservation efforts, is the only aquatic mammal inhabiting the Yangtze River. Overexploitation of Yangtze River resources has greatly damaged the river’s ecology, however, and fishery resources have declined markedly over recent decades. Similar to the baiji (*Lipotes vexillifer*), YFP are in danger of extinction if no protective measures are taken [1], despite the many urgent protection measures and policies that have already been implemented, including the establishment of natural reserves and ex-situ conservation efforts [2]. However, a lack of information about the seasonal distribution, behaviors, and population dynamics of YFP poses a major challenge for the conservation of this species in the Yangtze River [3]. Because traditional field surveys are time consuming and costly [4], there is an urgent need for more effective methods of monitoring YFP populations in the Yangtze system.

Environmental DNA (eDNA), generated from shed skin, defecation, and other biological processes, persists in the environment and serves as a record of a given species presence [5]. In aquatic environments, eDNA analysis has been found to be an economical, efficient, and sensitive tool, with the bonus that it is a non-invasive form of sampling [6]. eDNA methods have thus been widely applied for identifying species [7], investigating endangered species [8], tracking invasive species [4], and monitoring biodiversity [9]. However, in aquatic settings, most eDNA studies have focused on clear or calm freshwater systems [10]. Compared to research on YFP in the Tian e-Zhou National Nature Reserve [11], determining YFP presence via eDNA is likely to be more challenging in the Yangtze River system due to the river’s high turbidity and strong current, factors that tend to rapidly dilute eDNA. Furthermore, the high sediment loads of the samples can inhibit amplification if eDNA is of low quality and quantity [12]. As such, the effectiveness of eDNA methods depends on the effective capture of eDNA when concentrations are very low [13].

Here, we used eDNA analysis to investigate the distribution of YFP in the Yangtze River. Sampling sites were chosen based on both observed and suspected presence of YFP, and a detailed sampling protocol was developed to maximize eDNA recovery and minimize contamination. With the aid of qPCR, YFP distributions in both summer and winter were analyzed to better inform YFP conservation management in the Yangtze River system.

## Materials and methods

### Field surveys

**Fig 1:**
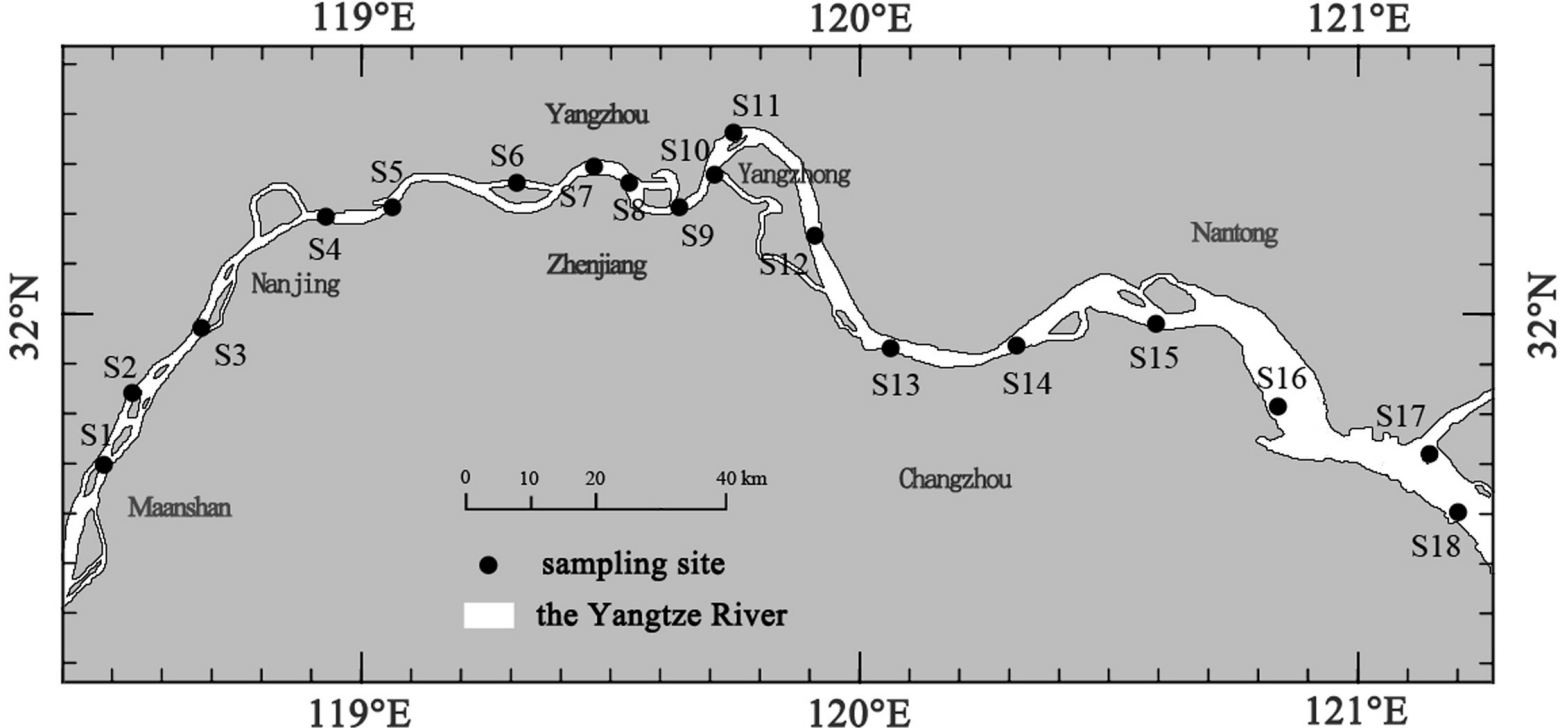
Locations of the summer field survey sampling sites (August 2017) generated by ArcGIS 10.0.

The YFP field survey was approved by the Ministry of Agriculture and Rural Area. A summer YFP field survey was carried out in the Yangtze River that extended from Xinshengzhou (31°46′17.48″N, 118°28′10.92″E) to the Dongfengxisha Reservoir (31°40′53.56″N, 121°11′19.47″E) in August 2017. Eighteen sites (S1–S18) were selected for sampling during this period (Fig 1). Visual observations were carried out using a telescope after the survey vessel reached the chosen site and had been stopped for 10 minutes; YFP were visually observed at 7 (S4, S7, S8, S10–S12, and S17) and not observed at 11 (S1–S3, S5, S6, S9, S13–S16, and S18) of the 18 sites (S1 Fig). However, the 11 sites were also suitable habitats for YFP, as they were less navigable for shipping vessels and supported rich food resources.

**Fig 2:**
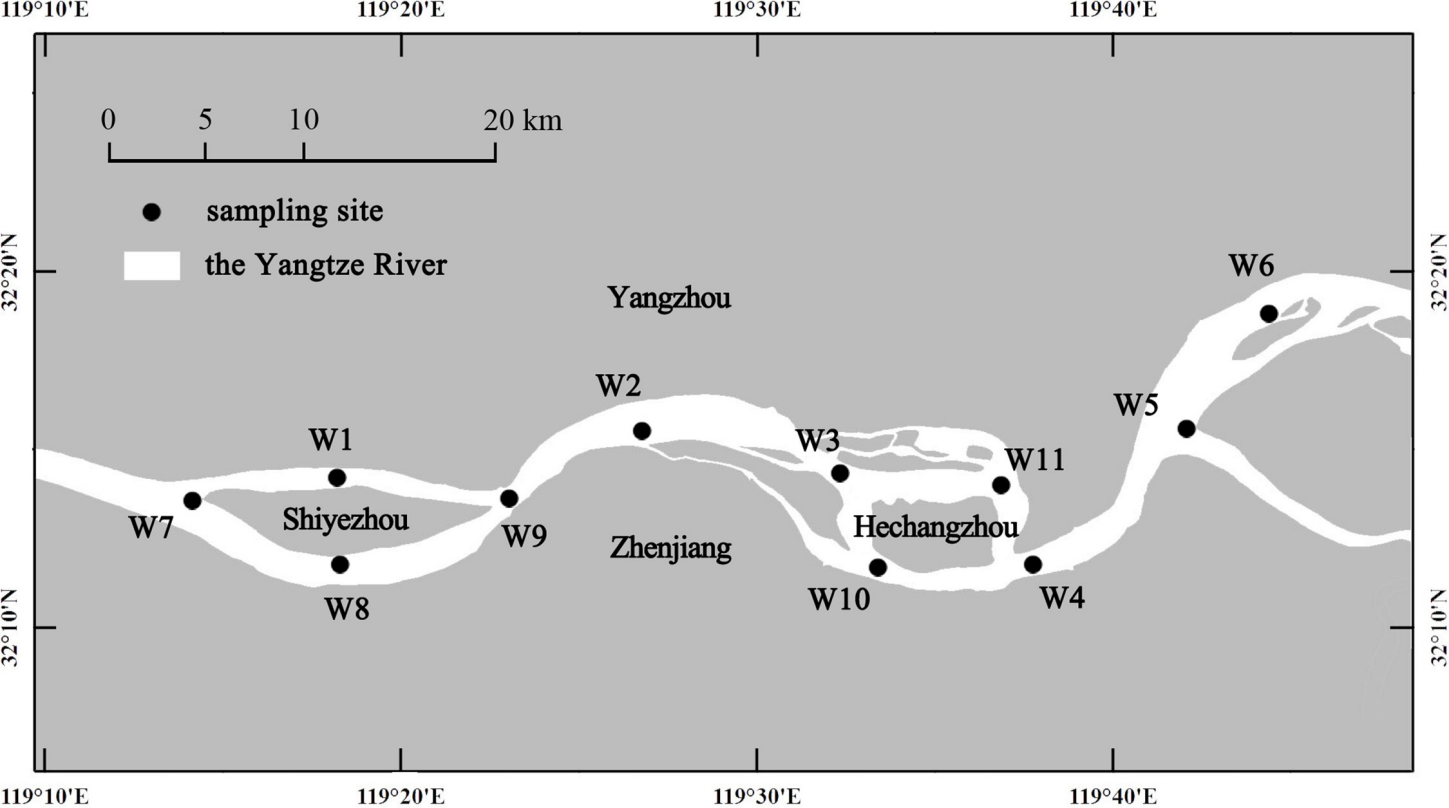
Locations of the winter field survey sampling sites (January 2018) generated by ArcGIS 10.0.

In January 2018, a winter field survey was carried out in the Zhenjiang Yangtze Dolphin Provincial Nature Reserve, extending from Shiyezhou (32°13′25.82″N, 119°14′24.71″E) to Yangzhong (32°19′1.28″N, 119°46′34.36″E). Eleven sampling sites (W1–W11) were selected for this field survey (Fig 2), six of which (W1–W6) were the same as visited in the summer field survey (S6–S11). Several groups of YFP were observed around three winter sampling sites (W4–W6), whereas the remaining sites (W1–W3 and W7–W11) were considered sites of possible inhabitation.

### Water sample collection and filtration

Once the survey vessel reached the chosen destination and had been stopped for 10 min, water samples were collected from below the surface (depths of 0.3–0.6 m) with new 1 L sealed plastic bottles. The sample containers were rinsed with 10% bleach solution, and then rinsed thoroughly with tap water and allowed to air dry prior to water sample collection. All filtration equipment was carefully rinsed with distilled water between filtration operations to prevent cross-contamination.

At each sampling site, we filtered six 1 L water samples, treated as replicates, followed by one 1 L negative control by replacing the environmental sample with distilled water, which was subsequently extracted and amplified. Sampling water was pre-filtered using disposable, medical-grade gauze to prevent large suspended particles from clogging the filter papers. In the pre-experiment, we selected the 5µm, 1µm and 0.45µm pore size nylon filter paper according to the reference. However, we found it will take us about 1h filtering 500ml water through 1um filter paper. So 5 µm pore size nylon filter paper with 47 mm diameter was selected. Water was filtered immediately on site by a vacuum pump. After filtering, we removed and rolled the filter with forceps, and then placed the filter into a sterile 2 ml microtube filled with 95% ethanol as a preservative. To avoid cross-contamination, every six microtubes from each sampling site were sealed in an individual labeled plastic bag and stored at -20°C until the time of DNA extraction. Water temperature, pH, and dissolved oxygen (DO) were recorded at each sampling site, as were filtration time and the weight increment of each filter paper.

### eDNA extraction

eDNA was extracted from the filter paper using a Water DNA Kit (Omega Bio-Tek, USA) in accordance with the manufacturer’s instructions. Incubation temperature was set at 90°C to lyse any bacteria, and 50 µL of elution buffer was applied to elute DNA from the column and concentrate the eDNA. All samples were then immediately stored at -20°C.

### Real-time PCR

A pair of specific primers was designed to amplify a 102 bp fragment of the mitochondrial D-loop sequence of YFP DNA according to the partial sequence of *Neophocaena phocaenoides asiaeorientalis* mitochondrial control region H1 (GenBank accession number: AY334099.1). The sequences of forward and reverse primers are (5′-TATGTCCACTAGCCCTTCATAACCATTA-3′) and (5′-AGATCATTATTTAGCTACCCCCACAAGC-3′), respectively. Quantitative real-time PCR was performed with a Takara TP800 Thermal Cycler Dice Real Time System (Takara, Dalian), and PCR was carried out in a total volume of 20 µL containing 2 µL of extracted template DNA, 0.8 µL of each primer (10 µM), 10 µL 2× SYBR Premix Ex *Taq*^TM^ II (Takara, Dalian), and 6.4 µL of ddH_2_O. The PCR conditions were as follows: 3 min at 95°C, 45 cycles of 10 s at 95°C, and 20 s at 60°C, followed by a final melt-curve stage. One positive control (plasmid template) and two negative controls (DNA extraction negative controls and qPCR negative controls) were included in every run, and all qPCR reactions were performed in triplicate.

Positive controls were amplified in every run, and the two negative controls were not; if two or more PCR replicates were amplified at the same melting temperature as that of the positive control, the water sample was recorded as positive, whereas if only one of three PCR replicates was amplified successfully, the water DNA extract was retested in another round of PCR. A sampling site was recorded as positive for YFP if one or more water samples from that site were determined to be positive.

### Establishment of a standard curve

The partial region of YFP mitochondrial D-loop was amplified with primers (F: 5′-AAGCTGGAATTCTTTATAAACTACTC-3′; R: 5′- AACTATCTGTATGATTTCATTATGGG-3′) according to the partial sequence of *Neophocaena phocaenoides asiaeorientalis* mitochondrial control region H1 (GenBank accession number: AY334099.1). PCR products were cloned into a pMD 18-T Vector (Takara, Dalian), and a dilution series of the plasmid containing 10^1^–10^7^ copies per µl were performed in qPCRs to establish a standard curve.

### Data analysis

We used general linear modeling (GLM) to determine whether the filtration time of each sample was related to abiotic features or weight increments. Levene’s test of equality of error variances was used to assess eDNA data for normal distribution, and a two-way mixed ANOVA was used to test for the effects of sites and seasons on YFP eDNA concentration. All statistical analyses were performed in SPSS 19.0 (SPSS Inc., Chicago, USA).

## Results

### Primers test and standard curve

**Fig 3:**
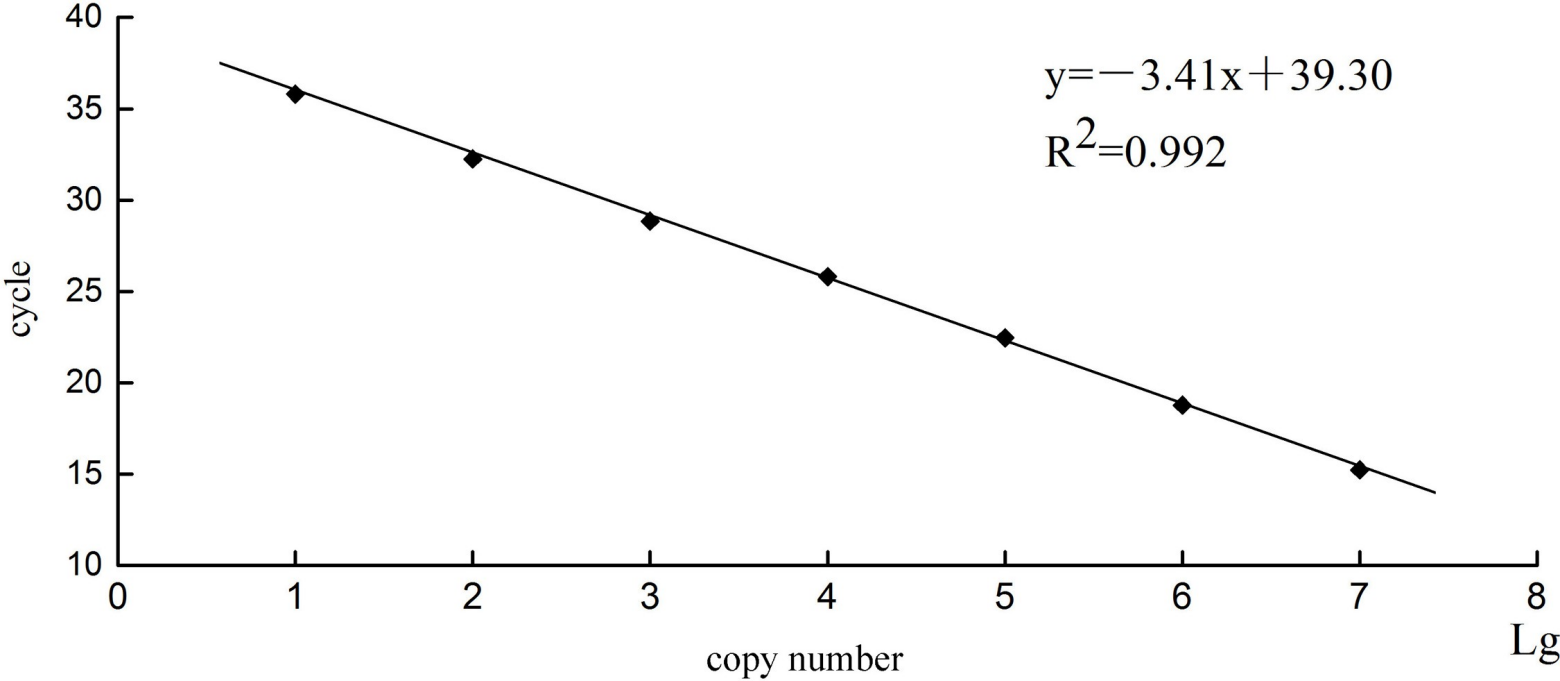
The standard curve of the YFP mitochondrial D-loop.

A standard curve of YFP D-loop for real-time qPCR was calculated with plasmid, as a linear regression (y = -3.41x+39.30, R^2^ = 0.992) with a PCR efficiency of 96% (Fig 3). Positive controls were tested using eDNA extracted from purse seine water collected from a site in the Xijiang Nature Reserve where one YFP lived temporarily; as expected, the water samples exhibited amplification. Negative controls were also tested and displayed no amplification. These results imply that our specific primers could be used for amplification and detection of eDNA under optimum qPCR conditions.

### Detection rate of eDNA and traditional field survey methods

It took 13.7–23.2 min and 9.6–16.4 min to filter 1 L water samples in summer and winter, respectively. Water temperature of the samples ranged from 22.3–28.2°C in summer and 9.3–10.7°C in winter; pH ranged from 8.47–9.15 in summer and 8.29–8.90 in winter; DO ranged from 6.42–9.77 mg/L in summer and 7.84–8.96 mg/L in winter; and the weight increment of each filter paper after filtering ranged from 0.134–0.647 g. No significant relationships were detected between filtration time and these abiotic features (*P*>0.05), although the weight increment increased significantly with filtration time in both summer (R^2^ = 0.72, *P*<0.001) and winter (R^2^ = 0.81, *P*<0.001) samples.

We found that analysis of eDNA had a higher detection rate than traditional field surveys for the presence of YFP in the Yangtze River. In samples collected during the summer field survey, we successfully detected YFP eDNA at seven sites (S4, S7, S8, S10–S12, and S17) where YFP were visually observed via telescope and an additional four sites (S2, S3, S9, and S15) of 11 sites where YFP were not observed but suspected to inhabit (Table 1). In samples collected during the winter field survey, we detected YFP eDNA at four sites (W3–W6) where YFP were visually observed and at an two (W2 and W11) of seven additional sites where YFP were not observed but suspected to inhabit (Table 1). We did not detect YFP eDNA in any of the eDNA extraction negative controls or the qPCR negative controls. Notably, YFP was detected both upstream and downstream of the Zhenjiang Yangtze Dolphin Provincial Nature Reserve, despite individuals never before visually observed in these areas during numerous previous field surveys.

**Table 1:** Detection results of YFP in field survey.

Sampling site	Number of individuals of each group visually observed	Results of qPCR	Results of detection
**S1**	0	0/18	negative
**S2**	0	18/18	positive
**S3**	0	16/18	positive
**S4**	2	10/18	positive
**S5**	0	0/18	negative
**S6**	0	0/18	negative
**S7**	3, 5	18/18	positive
**S8**	10, 4	18/18	positive
**S9**	0	18/18	positive
**S10**	3	14/18	positive
**S11**	4, 5	18/18	positive
**S12**	2	11/18	positive
**S13**	0	0/18	negative
**S14**	0	0/18	negative
**S15**	0	18/18	positive
**S16**	0	0/18	negative
**S17**	3, 4	17/18	positive
**S18**	0	0/18	negative
**W1**	0	0/18	negative
**W2**	0	14/18	positive
**W3**	2, 4	18/18	positive
**W4**	3, 4	18/18	positive
**W5**	2, 3	18/18	positive
**W6**	2	17/18	positive
**W7**	0	0/18	negative
**W8**	0	0/18	negative
**W9**	0	0/18	negative
**W10**	0	0/18	negative
**W11**	0	18/18	positive

### Influence of season on eDNA concentration

**Fig 4:**
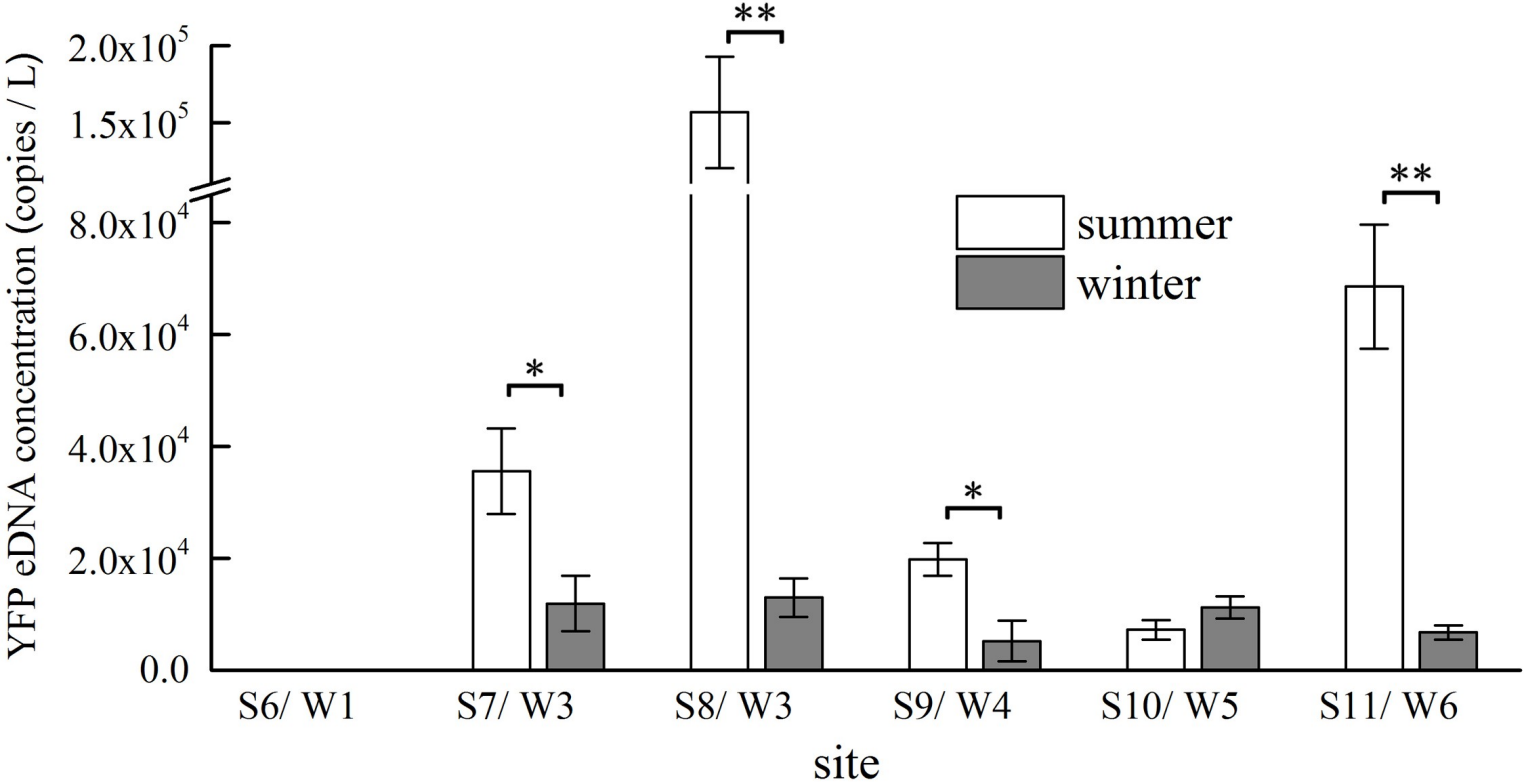
Comparison of YFP eDNA concentrations at six sites in summer and winter. * significant (*P*<0.05); ** highly significant (*P*<0.01).

We compared detection results for six sampling sites that were included in both the summer (S6–S11) and winter (W1–W6) sampling surveys; these sites were almost in precisely the same locations, based on GPS positions. All six sites tested positive for YFP eDNA except for the S6/W1 site. Of the five positive sites, there was a significant difference in YFP eDNA concentration between summer and winter, with the exception of the S10/W5 site (F_5, 30_ = 81.93, *P*<0.001). Although YFP was also observed in winter, significantly higher amounts of eDNA were detected in the summer samples than in the winter samples (Fig 4).

## Discussion

YFP is listed as a Class II protected species by the national government of China, and is the only mammal inhabiting the Yangtze River [14]. Because of its endangered status, restrictions are put on many traditional methods of investigation, such as marker and recapture approaches. In our observation-based field survey, it took at least eight observers, all with expertise in identifying the morphological traits of YFPs used to distinguish among individuals, an entire week to conduct observations of YFP; moreover, such surveys are entirely weather dependent. In contrast, only two researchers were needed to collect water samples, extract DNA extraction, and conduct qPCR over the same time period using eDNA techniques. For each sample, we used 5 µm pore size nylon filter papers to filter 1 L of pre-filtered water quickly, and for eDNA analysis, qPCR was used to measure the copy number of a YFP mitochondria fragment.

eDNA released into aquatic environments by individual animals is a continuous process but is not simply homogeneous, especially in areas of flowing water where eDNA particles precipitate slower than they do in still water [15]. Emissions associated with predation, reproduction, or other behaviors greatly increase the amount of eDNA released into aquatic environments [16]. However, hydrological mixing of eDNA emissions and animal activities contribute to relatively stable eDNA concentrations within a 50–450 m extent downstream [17]. In our study, eDNA concentrations in six replicate samples showed high levels of consistency over a 300 m stream reach and many sampling sites, especially in locations where more than three YFP were observed. We also found that the distance between the sampling site and the riverbank significantly influenced eDNA concentrations. Most of our sampling sites were ~700–1,000 m from the riverbank, but several were greater than 1,000 m from the riverbank due to a lower riverbed, where strong currents would disperse eDNA emissions rapidly and enhance YFP eDNA degradation, resulting in lower concentrations of or even undetected YFP eDNA. In the field survey, most of the observed YFP groups were located near the riverbank (~500 m). Taken together, our results suggest that YFP eDNA sampling sites in the Yangtze River should be no more than 200–500 m from the riverbank.

Previous research has clearly shown that eDNA concentration is strongly correlated with the target species distribution and environmental factors, such as season and particle size of suspended sediments [18–20]. Although eDNA degradation was slower in winter [21], high water temperatures promote physiological metabolism and enhance the intensity of activity of aquatic animals, resulting in higher levels of secretion or shedding [22]. We found that YFP eDNA concentration also varied between summer and winter, with eDNA concentrations higher in summer than in winter. As such, eDNA approaches will be more effective at detecting YFP distributions in the Yangtze in summer than in winter.

Detection of eDNA in muddy [23] and turbulent freshwater systems [5] suggests that eDNA analysis can be an appropriate and inexpensive tool for determining the presence and distribution of YFP in the Yangtze River. Our results indicate that the greater sensitivity of eDNA analysis makes it a more effective tool than traditional field surveys, as eDNA analysis revealed that the distribution of YFP may be larger than previously described, information that will inform future monitoring of YFP distribution in this river system.

## Supporting information

S1 Fig The Yangtze finless porpoise activity in the Yangtze River. (PDF)
