# Supplementary figures and images for: Investigating the distribution of the Yangtze finless porpoise in the Yangtze River using environmental DNA
Source: PLoS One. 2019 Aug 9;14(8):e0221120. doi: 10.1371/journal.pone.0221120 (PMC6688821; doi:10.1371/journal.pone.0221120)

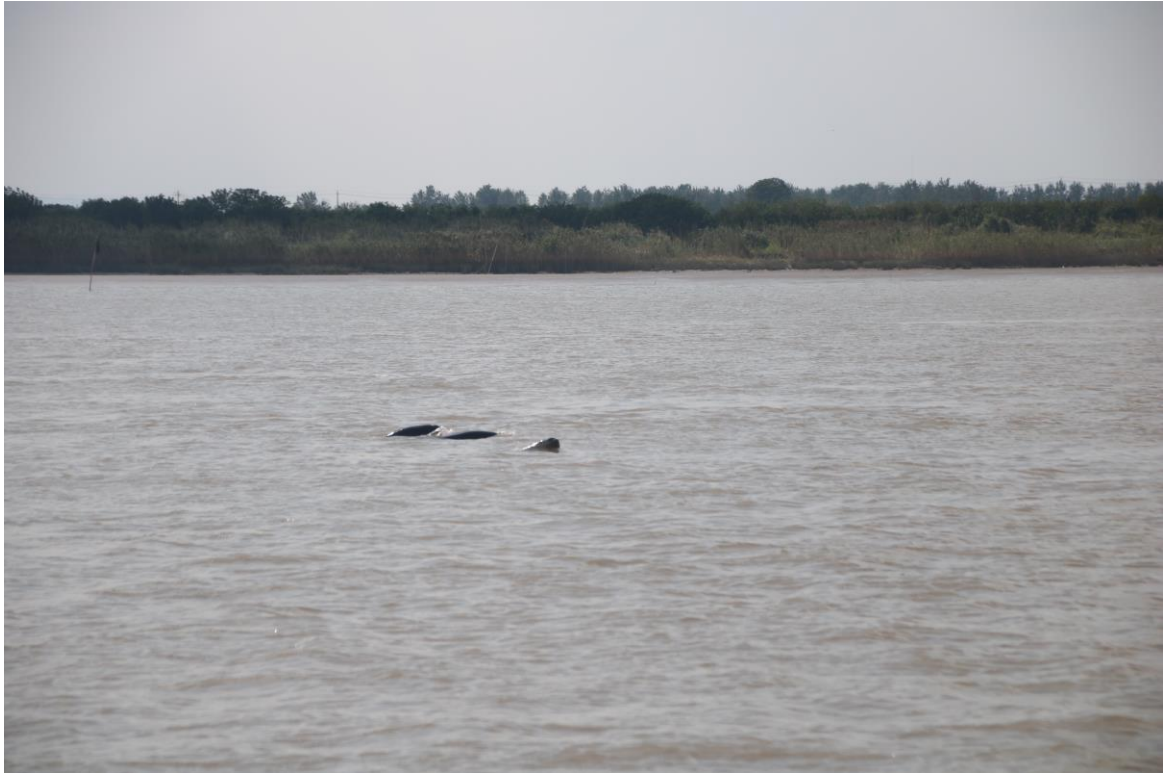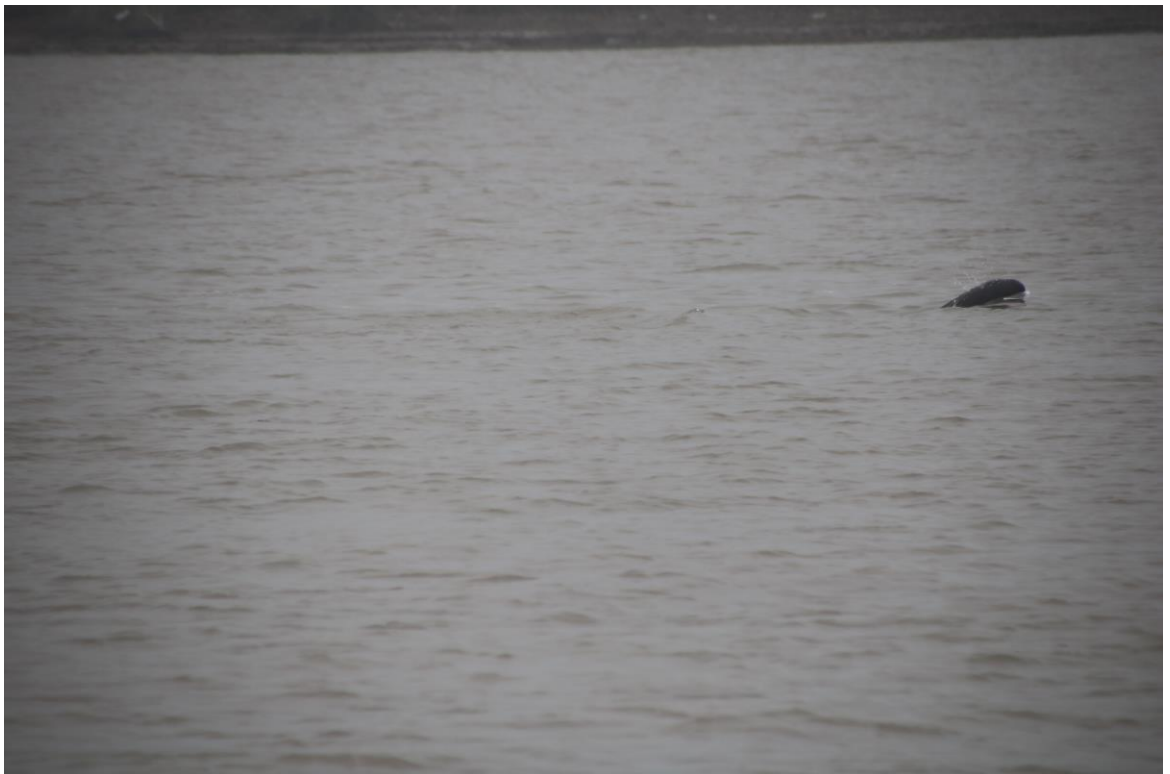

Supplement: S1 Fig — (PDF) [file pone.0221120.s001.pdf]
